# “Multisystem Inflammatory Syndrome in Children”-Like Disease after COVID-19 Vaccination (MIS-V) with Potential Significance of Functional Active Autoantibodies Targeting G-Protein-Coupled Receptors (GPCR-fAAb) for Pathophysiology and Therapy

**DOI:** 10.3390/children10121836

**Published:** 2023-11-22

**Authors:** Marius Schmidt, Steven Hébert, Gerd Wallukat, Rolf Ponader, Tobias Krickau, Matthias Galiano, Heiko Reutter, Joachim Woelfle, Abbas Agaimy, Christian Mardin, André Hoerning, Bettina Hohberger

**Affiliations:** 1 Department of Pediatrics and Adolescent Medicine, University Hospital Erlangen, 91054 Erlangen, Germanygastroenterologie.kinder@uk-erlangen.de (A.H.); 2 Berlin Cures GmbH, 13125 Berlin, Germany; 3 Department of Pediatrics and Adolescent Medicine, 95032 Hof, Germany; 4 Division of Neonatology and Pediatric Intensive Care, Department of Pediatrics and Adolescent Medicine, University Hospital Erlangen, 91054 Erlangen, Germany; 5 Department of Pathology, University Hospital Erlangen, 91054 Erlangen, Germany; 6 Department of Ophthalmology, University Hospital Erlangen, 90766 Erlangen, Germany; 7 German Center for Immunotherapy, University Hospital Erlangen, Friedrich-Alexander-University Erlangen-Nuremberg, 91054 Erlangen, Germany

**Keywords:** COVID-19, SARS-CoV-2, MIS-V, GPCR-fAAb, MIS-C, PIMS

## Abstract

Background: An infection with SARS-CoV-2 can trigger a systemic disorder by pathological autoimmune processes. A certain type of this dysregulation is known as Multisystemic inflammatory syndrome in children (MIS-C). However, similar symptoms may occur and have been described as Multisystemic inflammatory syndrome after SARS-CoV-2 Vaccination (MIS-V) following vaccination against SARS-CoV-2. We report the case of a 12-year-old boy who was identified with MIS-C symptoms without previous SARS-CoV-2 infection after receiving two doses of the Pfizer–BioNTech COVID-19 vaccine approximately one month prior to the onset of symptoms. He showed polyserositis, severe gastrointestinal symptoms and, consequently, a manifestation of a multiorgan failure. IgG antibodies against spike proteins of SARS-CoV-2 were detected, indicating a successful vaccination, while SARS-CoV-2 Nucleocapsid protein antibodies and SARS-CoV-2 PCR were not detected. Several functional, active autoantibodies against G-protein-coupled receptors (GPCR-fAAb), previously associated with Long COVID disease, were detected in a cardiomyocyte bioassay. Immunosuppression with steroids was initiated. Due to side effects, treatment with steroids and later interleukin 1 receptor antagonists had to be terminated. Instead, immunoadsorption was performed and continued with tacrolimus and mycophenolic acid therapy, leading to improvement and discharge after 79 days. GPCR-fAAb decreased during therapy and remained negative after clinical curing and under continued immunosuppressive therapy with tacrolimus and mycophenolic acid. Follow-up of the patient showed him in good condition after one year. Conclusions: Infection with SARS-CoV-2 shows a broad and severe variety of symptoms, partly due to autoimmune dysregulation, which, in some instances, can lead to multiorgan failure. Despite its rarity, post-vaccine MIS-C-like disease may develop into a serious condition triggered by autoimmune dysregulation. The evidence of circulating GPCR-fAAb and their disappearance after therapy suggests a link of GPCR-fAAb to the clinical manifestations. Thus, we hypothesize a potential role of GPCR-fAAb in pathophysiology and their potential importance for the therapy of MIS-C or MIS-V. However, this observation needs further investigation to prove a causative correlation.

## 1. Introduction

SARS-CoV-2 is a virus from the family of Coronaviridae. A SARS-CoV-2 infection primarily causes symptoms similar to the common flu, with patients presenting with fever, respiratory disorder and myalgia [1,2,3,4,5]. Soon, it was discovered that SARS-CoV-2 also affects the immune system by triggering hyper-inflammatory reactions, which eventually may cause a systemic illness called Multisystemic inflammatory syndrome in children, i.e., “MIS-C” [6,7,8,9,10]. However, vaccination against SARS-CoV-2 shows potential interactions with the immune system that may, in rare cases, cause similar disorders of autoimmune reactions and disorders [11,12]. This autoimmune disorder was soon to be described as Multisystemic inflammatory syndrome after SARS-CoV-2 Vaccination—“MIS-V” [13,14]. 

Here, we highlight a case of a 12-year-old boy who, even though not verifiably previously infected with SARS-CoV-2, showed a wide range of symptoms similar to MIS-C, resulting in the necessity of initiating an immunosuppressive therapy. 

## 2. Case Presentation

### Symptoms at First Visit

A 12-year-old male adolescent was admitted at an external pediatric facility outside the metropolitan area of Nuremberg–Erlangen with fever first occurring several days after receiving the second injection of the Pfizer–BioNTech COVID-19 vaccine. He received the first vaccination 47 days prior to admission at our hospital and the second dosage 21 days prior to admission at our hospital. A timeline of the case can be seen in Supplementary Materials Figure S1. Initially, he showed symptoms of diarrhea and a respiratory infection, which was later also exhibited transitorily in the patient’s mother. The results of stool testing for viral and bacterial pathogens were negative. Additionally, the patient presented with leukopenia and thrombopenia. Red blood cells (RBCs) were normal, with an absence of schistocytes. The patient showed elevated liver enzymes, lactate dehydrogenase, pancreatic lipase and creatinine. Urine analysis and quantity of urine were normal.

Chest X-rays showed no expedient findings. Initial abdominal ultrasound revealed normal liver and spleen size. However, the gallbladder wall was thickened and showed signs of hydrops. Over time, a small amount of ascites developed that was not eligible for drainage. ECG and long-term ECG, as well as a transthoracic echo, revealed no signs of myocarditis; in particular, coronary arteries were unaffected. Ophthalmologic examination on day 4 showed mild conjunctivitis but no retinal vasculitis.

Hepatitis serology indicated a previously solved but not acute infection with the hepatitis E virus. Even though a vaccination was documented, no antibodies against the hepatitis B surface antigen could be assessed. PCRs for CMV, EBV, hepatitis B, C, D and E were also negative. For two years, the patient had been showing recurrent but self-limiting symptoms of cheilitis, which generally aggravated during winter. 

**Figure 1:**
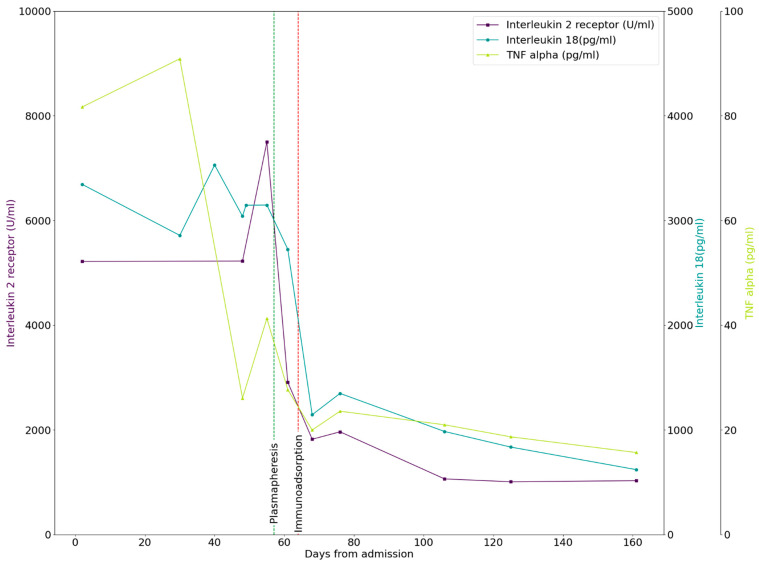
Longitudinal course of inflammatory cytokines. Serum concentrations of inflammatory cytokines TNF-alpha Tumor necrosis factor (TNF-alpha, light green), interleukin-2 receptor (IL-2 receptor, purple) and interleukin 18 (IL-18, dark green) are shown over time. Time of surveillance for this patient was from June 2021–January 2022. Time points of plasmapheresis and immunoadsorption are depicted in green or red dotted vertical lines, respectively.

One week after the initial presentation, the patient was transferred to the University Hospital Erlangen. For further discussion, this admission will be referred to as day 1. Laboratory values throughout the clinical progress are demonstrated in Table S1 of the Supplementary Materials. The boy exhibited mild prerenal failure with a creatinine increase to 1.0 mg/dL (reference scale 0.39–0.77 mg/dL). Coagulation was infringed with a Prothrombin time (PT) of 59% (reference scale 78–105%), with a Partial thromboplastin time (PTT) in the normal range. Prior to this, the patient was given oral phytomenadione, which did not result in any significant improvement in coagulation. Urine analysis and quantity of urine showed normal laboratory values, which remained relatively unchanged compared to the prior externally analyzed samples. Troponin I and B-type natriuretic peptides were in the normal range. Abdominal ultrasound showed hepatosplenomegaly and pericardial, pleural and peritoneal effusions, indicating polyserositis. Inflammatory factors such as Tumor necrosis factor-alpha (TNF-alpha), soluble interleukin-2 receptor (IL-2 receptor) and interleukin 18 (IL-18) were significantly increased (Figure 1). 

In the physical examination, the patient showed several muco-cutaneous lesions, predominantly a cheilitis. In blood cultures and oral swaps, Candida krusei was observed. Regarding the pronounced lesions, we see these as a possible portal of entry for the fungemia. Additionally, the patient had braces, which were removed when the clinical condition of the patient stabilized. As a consequence, therapy with liposomal Amphotericin B as well as Ampicillin/Sulbactam was initiated. Because of the persistent liver failure and the ultrasonographic picture of a juvenile metabolic associated dysfunction-associated steatotic liver disease (MASLD), an extensive diagnostic for metabolic disorders, such as storage disorders like Lysosomal Acid Lipase Deficiency (LALD), Acid Sphingomyelinase Deficiency, Alpha-1 antitrypsin deficiency and Wilson’s disease, was performed. The results remained negative. Secondary findings showed unexpected warm-reacting antibodies against erythrocytes (Anti-Jk^a^). 

**Figure 2:**
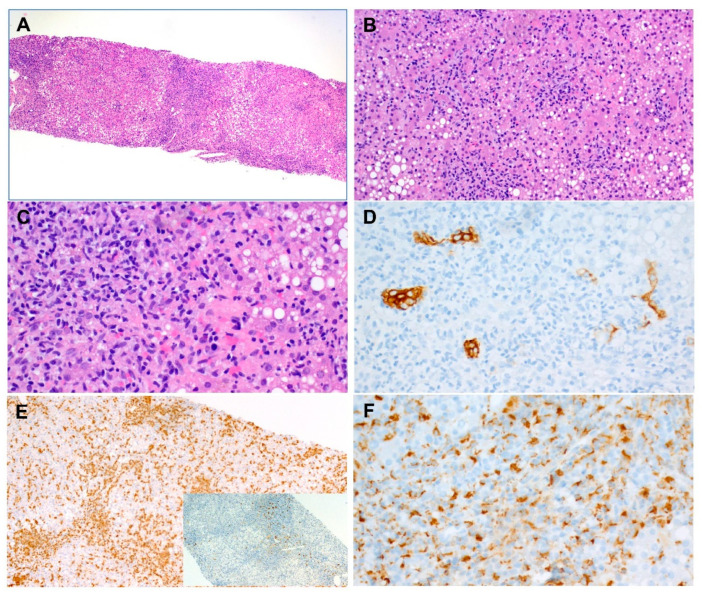
Liver histology. (**A**) Overview showing highly cellular portal and lobular inflammatory infiltrate. Note: prominent steatosis (metabolic dysfunction-associated fatty liver disease, MAFLD) (×50). (**B**) Higher magnification of the lobular inflammation (×100). (**C**) Higher magnification of the portal inflammation (×200). (**D**) CK7 immunostain highlighting destroyed and atrophic bile ductile epithelium (×200). (**E**) Main image: extensive CD3 positive lymphocytes. Sub-image: very sparse CD20-positive B-lymphocytes (×100). (**F**) Prominent CD68-positive macrophages (×200).

On day 7 after admission to our clinic, a combined liver and bone marrow biopsy was performed. Histologically, the liver showed inflammatory infiltrations, mainly with evidence of activated macrophages, which could be detected both in the portal field and in the lobular area, as well as cholangitis (Figure 2). 

Additionally, extensive MAFLD was histopathologically seen and evaluated as co-existing independently from the inflammation process. Extended and repeated testing for autoimmune hepatitis via autoantibody testing solely revealed an unspecific elevation of antinuclear antibodies (ANAs), while lupus diagnostic parameters (anti-dsDNA, lupus anticoagulants) remained repeatedly negative. The patient did not have a history of recurring infections. For immunodeficiency testing, we performed B-cell differentiation twice. Initially, B- and T-lymphocytes were lightly reduced. One month later, both cell lines showed normal T-lymphocytes and lightly elevated B-lymphocytes. Antibodies against tetanus and diphtheria showed normal values and, therefore, appropriate immune response. IgG subclasses were in the normal range.

IgG antibodies against the spike protein of SARS-CoV-2 were detected, indicating a successful vaccination. However, findings of SARS-CoV-2 Nucleocapsid protein antibodies were negative. 

Bone marrow histology excluded a macrophage activation syndrome (MAS). Moreover, it showed no signs of malignity; therefore, an immunosuppressive therapy with steroids was initiated. On day 10, pancreatic lipase was increasing. Assuming a side effect, steroid treatment was stopped, and subsequently, Anakinra (interleukin-1 receptor antagonist) was administered (100 mg twice a day) immediately. Under this treatment, the patient significantly improved clinically and was released in good condition on day 23. 

**Figure 3:**
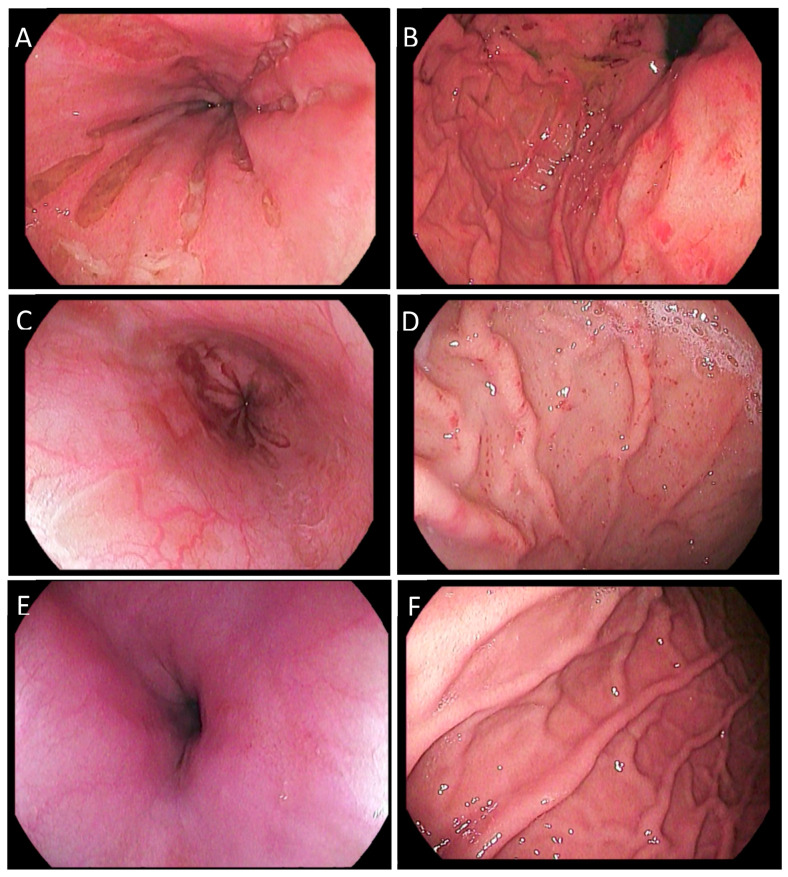
Endoscopic findings of esophagus (left) and gastric corpus (right) at decision of initiating plasmapheresis and immunoadsorption (**A**,**B**). Two months later (**C**,**D**), an amelioration of the esophageal ulcera and gastric erosions could be observed. Mucosal healing was documented 9 months later (**E**,**F**).

However, soon after, on day 33, the patient was readmitted due to clinical decline. The boy showed a deterioration of his general condition, presented with fever, felt tired and suffered from a lack of appetite with persistently increasing inflammation parameters (Figure 1). Therefore, the therapy of Anakinra was altered to a total of two doses of Canakinumab (150 mg administered s.c. on day 34 and day 43). On day 35, the patient was shifted to the ICU due to a hemodynamically unstable situation without any bacterial findings in the blood culture. An Angio-CT on day 36 showed no evidence of caliber fluctuations, wall thickening or stenosis and no evidence of vasculitis. Here, an intensified therapy with steroids and intensive intravenous fluid was administered, from which the patient recovered within 48 h. Gastroscopy, performed on the same day, showed circular multiple streaky ulcerative-erosive punched-out-looking lesions in the distal esophagus, as well as mucosal erosions in the stomach (Figure 3). Histopathology showed no evidence of dysplasia but signs of chronic inflammation.

On day 37, several functional active GPCR-fAAb that have been previously associated with Long COVID disease were detected. Determination of functional, active autoantibodies targeting G-protein-coupled receptors (GPCR-fAAb) was performed with a cardiomyocyte bioassay as previously described by Wallukat et al. at Berlin Cures GmbH laboratory (Berlin, Germany) [15]. 

The chronological sequence and list of GPCR-fAAb can be seen in Table S2 of the Supplementary Materials. Another ophthalmologic examination on day 43 no longer found signs of conjunctivitis. However, retinal vasculitis, in contrast to the initial examination at admission, was found. Local steroid therapy was initiated for 5 days. Repeated examination on day 53 showed no finding of vasculitis. During the night of day 49, the patient once again showed clinical deterioration, leading to a septic shock. Blood culture confirmed an infection with staphylococcus haemolyticus, with consecutive treatment with vancomycin, initiated on day 50. The patient recovered and could return to the regular ward 24 h later. Considering the persistently ongoing multisystemic inflammation process with a persisting reduced clinical condition, we decided to initiate extracorporeal apheresis therapy for a total of five times from day 57 until day 62, followed by immunoadsorption (IA) from day 64 to day 75 (TR350 Immusorba/Diamed (Asahi Kasai) with plasma filter Plasmaflo OP-05W(L) (Asahi Kasai)) for a total of six times, during which the GPCR-fAAb were eliminated. Subsequently, the application of one-time 2 g/kg polyvalent intravenous immunoglobulins (IVIG) was performed. 

Additionally, we began treatment with tacrolimus (targeted trough level 8–10 ng/mL) and mycophenolic acid (targeted AUC 60). Upon initiating this therapy, the patient showed clinical improvement, a reduction of polyserositis and a subsequent decrease in inflammation parameters. The boy was released from the hospital on day 79 and presented again at two weeks (day 106), two months (day 125) and three months (day 161) for routine follow-up. During this follow-up period, he was in good general condition and displayed normal blood cell counts (Supplementary Table S1). GPCR-fAAb remained negative in serum samples for several months. However, in April of 2022 (day 251), we saw the reappearance of several GPCR-fAAb (Supplementary Table S2) even though the clinical condition of our patient remained good, besides recurrent abdominal pain. Though symptoms of cheilitis were persistent during both clinical stays, mucosal status had normalized. Follow-up endoscopy showed healing of the distal esophagus as well as the stomach. In addition to the immunosuppressive therapy regime consisting of tacrolimus and mycophenolic acid (MPA), tacrolimus dosage was adjusted to maintain blood levels stable between 4 and 7 ng/mL. MPA dosage remained unchanged. Omega-3 acid ethyl esters (one gram bid) were started with the intention of lowering the risk for an autoimmune relapse [16,17,18]. 

## 3. Discussion

SARS-CoV-2 predominantly causes symptoms of the respiratory tract but also may affect multiple organ systems, which can lead to various, and in some cases severe, symptoms, including organ failure [19,20,21,22,23,24]. A certain type of this systemic disease in children is known under the name of MIS-C [25,26,27,28]. In a systematic review and meta-analysis, Santos et al. described the most prevalent symptoms of MIS-C as fever and gastrointestinal symptoms including abdominal pain, skin manifestations and non-purulent conjunctivitis. They also highlight the similarities between MIS-C and Kawasaki disease [29]. Regarding the WHO definition of MIS-C, a fever of three or more days combined with elevated inflammation markers is required. Additionally, two or more symptoms have to be fulfilled [30]. The patient in our case report showed symptoms shortly after receiving the second dosage of the Pfizer–BioNTech COVID-19 vaccine. It is not certain whether they had developed symptoms already after the first dosage or after the second administration. There are reports of children showing symptoms after receiving only one dose of vaccination with the Pfizer–BioNTech COVID-19 vaccine [31]. Our patient suffered from oral muco-cutaneous lesions, conjunctivitis and diarrhea, as well as gastrointestinal lesions. Hence, a similarity with MIS-C can be postulated. However, there was no evidence of a prior infection with SARS-CoV-2, neither anamnestically nor serologically.

Over the last years, it was discovered that SARS-CoV-2, similar to other viruses like EBV or CMV, can induce the production of autoimmune antibodies due [32,33] to similarities in the genetic sequence between SARS-CoV-2 and humans, resulting in molecular mimicryKlicken oder tippen Sie hier, um Text einzugeben. [34,35]. Keka-Sylaj et al. describe a case of a 16-year-old adolescent suffering from MIS-C like symptoms following vaccination with Pfizer-BioNTech COVID-19 vaccine [36]. The patient’s main symptoms included hemodynamic instability due to infringed heart function with elevated cardiac enzymes. Eventually, administration of catecholamines was necessary. Since the patient was fulfilling MIS-C criteria, she was given corticosteroids and immunoglobulins, which led to a quick decrease in inflammation markers, while inotropic support was still required for ten days. Interestingly, while nasopharyngeal SARS-CoV-2 PCR was negative in contrast to our case, SARS-CoV-2 Nucleocapsid protein antibodies could be detected, however, only at a low level. 

Nune et al. described a similar case about a 44-year-old woman showing symptoms of MIS-A, the adult form of MIS-C, after being previously vaccinated with the Pfizer–BioNTech COVID-19 vaccine [13]. Core symptoms of arterial hypotension, fever and reduced kidney function could be seen, similar to our case. However, unlike our patient, the adult woman showed elevated troponin-T and had tenderness in several areas of her body. The woman was eventually treated with intravenous methylprednisolone, which led to a fast recovery of the patient’s clinical condition as well as a decrease in inflammation markers. An intensified immunosuppressive therapy or immunoadsorption, which was necessary in our case, was not performed. It has to be noted that, in our case, therapy with steroids could not be continued due to rising lipase, which was possibly a side-effect of steroid treatment. However, we saw a rather weak response of our patient’s clinical condition and inflammation markers to steroids not until extended therapy with tacrolimus and mycophenolic acid and immunoadsorption was begun. Since the symptoms seemed to be originated in the vaccination, Keka-Sylaj et al. and Nune et al. called it MIS-V, in which V stands for vaccination in order to emphasize the origin of the pathogenesis. Interestingly, it was seen that the severity and complexity of MIS-V symptoms seem to depend on the time between vaccination and manifestation of MIS-V. It can be hypothesized that a longer contact of the immune system with SARS-CoV-2 spike protein may induce an enhanced immune response, resulting in a stronger clinical manifestation, the longer the time between vaccination and onset of disease, potentially with a peak 1–2 months after SARS-CoV-2 vaccination [37]. 

MIS-V may be a potential mechanism for triggering the onset of an autoimmune disease in putatively genetically susceptible individuals [38,39,40]. An analysis of over 3 million patients with positive SARS-CoV-2 PCR showed a higher incidence of autoimmune disease following the infection in comparison with negative tested controls [41].

If you compare our case with other case reports of MIS-V like the ones described above or the case report of Karatzios et al. about two children, of which one also had predominantly heart-affecting symptoms [31], the fact that our patient needed intensified immunosuppressive therapy stands out. While the administration of steroids, IVIG and, in some cases, acetylsalicylic acid is normally enough to start recovery, this was not sufficient in the presented case. Thus, immunoadsorption was started, but it was not described in recent case reports of MIS-V.

Further research aimed for a more precise categorization of those autoantibodies. Wallukat et al. reported that autoantibodies of a certain group (GPCR-fAAb) were observed in patients’ sera suffering from symptoms following an infection with SARS-CoV-2 (i.e., Long COVID). Moreover, an association with some of these antibodies and diseases like multiple sclerosis was seen [15]. Interestingly, the removal of those GPCR-fAAb via immunoadsorption seemed to result in an improvement of the respective symptoms. Thus, GPCR-fAAb are assumed to be one of the mechanisms resulting in multisystemic symptoms occurring during or after an infection with SARS-CoV-2 [15]. Hébert et al. also suggested a connection between the appearance of those autoantibodies and MIS-C since the antibodies could be found in a patient of their cohort of children affected by MIS-C in the metropolitan area of Nürnberg–Erlangen [42].

To our astonishment, in our patient, five of the seven GPCR-fAAb described by Wallukat et al. could be measured by a cardiomyocyte bioassay. A sixth one was weakly positive. Parallel to the above-mentioned cases, immunoadsorption resulted in a substantial decrease of GPCR-fAAb in our patient’s serum and, finally, disappearance. From the moment of immunoadsorption, we saw clinical improvement in our patient. The lasting diminishment of GPCR-fAAb and absence of recreation of GPCR-fAAb we saw as a result of the immunosuppressive therapy commenced immediately after immunoadsorption. Based on the parallels of the clinical course of our patient and the serologic regression of GPCR-fAAb, a pathophysiological causality of GPCR-fAAb and clinical manifestation in our patient can be hypothesized. 

Our report shows parallels with Buchhorn et al.’s findings of GPCR-fAAb in an 18-year-old adolescent fulfilling the criteria of MIS-C after being vaccinated a second time with the Pfizer–BioNTech COVID-19 vaccine [43]. The patient suffered from mild elevation of cardiac enzymes whilst showing pericardial effusion. An additional patient described in Buchhorn et al.’s report was a thirteen-year-old girl suffering from Hashimoto thyroiditis with positive thyroid peroxidase antibodies (Anti-TPO) as well as atrioventricular block, which required pacemaker implantation [43]. Prior to vaccination with Pfizer–BioNTech COVID-19 vaccine, the girl already showed GPCR-fAAb. After receiving the vaccination, there was an increase in GPCR-fAAb and Anti-TPO, which progressed after the second vaccination. Interestingly, while GPCR-fAAb returned, Anti-TPO remained high. Of note, pacemaker activity increased during the period with elevated GPCR-fAAb.

We can consider an analogy to the SARS-CoV-2-triggerred post-viral Post-COVID Syndrome (PCS): patients with PCS showed (I) seropositivity of GPCR-fAAb (exemplarily β2-fAAb, AT1-fAAb and ETA-fAAb target vasoactive receptors) [15] and (II) a restricted capillary microcirculation (exemplarily in the retina, measured by OCT-angiography, OCT-A) [44,45]. Of interest, GPCR-fAAb were observed to show a clinical link to capillary microcirculation [46]. 

Based on the idea that GPCR-fAAb impair capillary microcirculation, a successful healing attempt was carried out in a patient with glaucoma and PCS by neutralizing GPCR-fAAbs (by an aptamer BC007, Berlin Cures GmbH), increasing capillary blood flow and consecutively ameliorating the PCS symptoms [47].

Of special interest for the presented case is the aspect that a seropositivity of GPCR-fAAb does not trigger a cellular effect independently. Yet, cellular surrounding seems to determine the functional cellular effect of GPCR-fAAb. This hypothesis is supported by data from Lukitsch et al. Functional active AT-1 Receptor AAbs showed a constriction of the renal arteries only in ischemic or inflammatory vessels. This is attributed to different conformations of the receptor, influenced by, e.g., ischemia or inflammation [48]. In addition, the degranulation of mast cells can be influenced by α1-fAAbs and AT1-fAAbs itself, contributing to a potential inflammatory circle [49]. 

Thus, we assume that GPCR-fAAb can act as a pathogenic factor in cells of the inflammatory vessels in PIMS. After the elimination of GPCR-fAAb by immunoadsorption, the inflammatory component was improved: post-IA, the levels of IL-6, CRP, BKS and IL-18 were within normal ranges. Only low levels of TNF-α and soluble IL2-receptor, a marker of Th1-response, were observed in the sera of the presented case. This cellular inflammatory recovery was accompanied by an amelioration of the PIMS’s symptoms. Thus, we hypothesize that elimination of the GPCR-fAAb by IA, even though being of temporary nature, enables the human body to recover from the cellular hyperstimulation with receptor desensitization. If the inflammatory component ameliorates within this ‘GPCR-fAAb free period’, even a relapse of GPCR-fAAb is not able to act pathogenic due to the ‘missing’ environmental cofactors.

This report might provide some thought that vaccinations targeting SARS-CoV-2 could potentially increase the risk of an onset of autoimmune disease and amplify the preexisting activity of the autoimmune mechanism—certainly with co-existing pre-disponing factors. While cardiac symptoms are a major part of the clinical picture of MIS-C, respectively, MIS-V, a key role of GPCR-fAAb found by us and Buchhorn et al. seems to be reasonable since their target structures are especially situated in cardiac tissue. Remarkably, our patient only suffered from mild cardiac symptoms. The reason for this varying clinical picture is yet to be clarified.

Further studies in patients suffering from illness after SARS-CoV-2 vaccination are therefore indicated. 

## 4. Conclusions

In summary, we described the case of an adolescent showing symptoms of autoimmune dysregulation following a vaccination against SARS-CoV-2. Multiple organ systems were affected, which led to, among other issues, liver failure and altered blood coagulation due to the dysregulation of the immune system. Intensified immunosuppressive therapy was eventually necessary to finally introduce remission. More information about the potential dysregulation of the immune system caused by SARS-CoV-2 vaccination is necessary. 

## Data Availability

The data presented in this study are available on request from the corresponding author, as anonymously documented data sets from clinical data systems of all participating hospitals. The data are not publicly available due to ethical restrictions on the personal data of patients.

## Supplementary Materials

The following supporting information can be downloaded at: https://www.mdpi.com/article/10.3390/children10121836/s1.

## Abbreviations

MIS-C, Multisystem inflammatory syndrome in children; GPCR-fAAb, functional active autoantibodies targeting G-protein-coupled receptors; IVIG, intravenous immunoglobulins; RBCs, red blood cells; CMV, cytomegalovirus; EBV, Epstein–Barr virus; AST, aspartate transaminase; ALT, alanine transaminase; GGT, gamma-glutamyltransferase; LDH, lactate dehydrogenase; PT, Prothrombin time; PTT, Partial thromboplastin time; TNF-alpha, TNF-alpha Tumor necrosis factor; IL-2 receptor, interleukin-2 receptor; IL-18, interleukin 18; ANAs, antinuclear autoantibodies; IA, immunoadsorption; MPA, mycophenolic acid; MIS-A, Multisystem inflammatory syndrome in adults; MIS-V, Multisystem inflammatory syndrome after SARS-CoV-2 vaccination; Anti-TPO, thyroid peroxidase antibodies; PCS, Post-COVID Syndrome.
