# Supplementary figures and images for: “Multisystem Inflammatory Syndrome in Children”-Like Disease after COVID-19 Vaccination (MIS-V) with Potential Significance of Functional Active Autoantibodies Targeting G-Protein-Coupled Receptors (GPCR-fAAb) for Pathophysiology and Therapy
Source: Children (Basel). 2023 Nov 22;10(12):1836. doi: 10.3390/children10121836 (PMC10741397; doi:10.3390/children10121836)

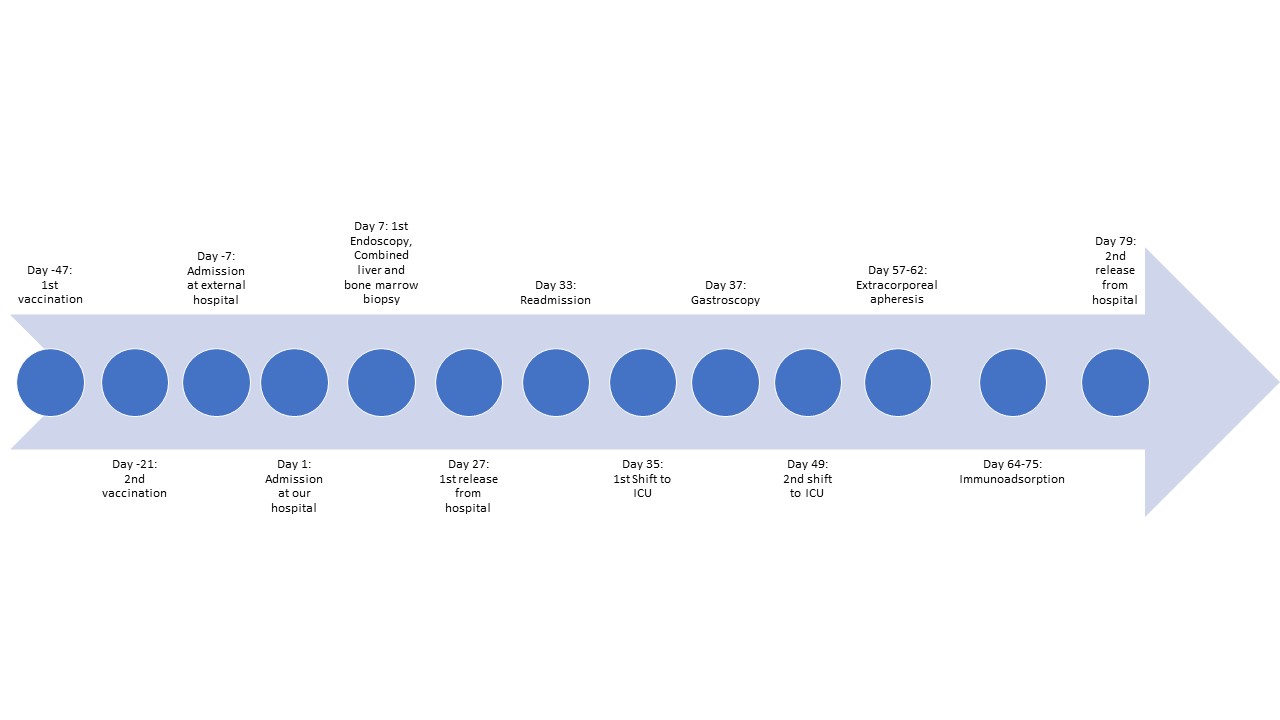

Supplement: Supplementary file 1 [file children-10-01836-s001.zip › Supplement S3 (Timeline)-Figure S1.jpg]
